# A functional variant of TLR10 modifies the activity of NFkB and may help predict a worse prognosis in patients with rheumatoid arthritis

**DOI:** 10.1186/s13075-016-1113-z

**Published:** 2016-10-04

**Authors:** Silvia Torices, Antonio Julia, Pedro Muñoz, Ignacio Varela, Alejandro Balsa, Sara Marsal, Antonio Fernández-Nebro, Francisco Blanco, Marcos López-Hoyos, Víctor Martinez-Taboada, Jose L. Fernández-Luna

**Affiliations:** 1 Servicio de Reumatología, Hospital Universitario Marques de Valdecilla-Instituto de Investigación Valdecilla (IDIVAL), Avenida Valdecilla s/n, 39008 Santander, Spain; 2 Unidad de Genética, Hospital Universitario Marques de Valdecilla-Instituto de Investigación Valdecilla (IDIVAL), Avenida Valdecilla s/n, 39008 Santander, Spain; 3 Rheumatology Research Group, Vall d’Hebron Research Institute, 08035 Barcelona, Spain; 4 Gerencia Atención Primaria, Servicio Cántabro de Salud, 39011 Santander, Spain; 5 Instituto de Biomedicina y Biotecnología de Cantabria, Universidad de Cantabria-CSIC, 39011 Santander, Spain; 6 Servicio de Reumatología, Hospital Universitario La Paz, 28046 Madrid, Spain; 7 Unidad de Reumatología, Instituto de Investigación Biomédica de Málaga, Hospital Universitario de Málaga, Universidad de Málaga, 29010 Málaga, Spain; 8 Departamento de Reumatología, Hospital Universitario A Coruña, 15006 A Coruña, Spain; 9 Sección de Inmunología, Hospital Universitario Marques de Valdecilla-Instituto de Investigación Valdecilla (IDIVAL), Avenida Valdecilla s/n, 39008 Santander, Spain; 10 Facultad de Medicina, Universidad de Cantabria, 39011 Santander, Spain

**Keywords:** TLR10 variant, NFkB, Rheumatoid arthritis, Infliximab

## Abstract

**Background:**

Toll-like receptor (TLR) family members are key players in inflammation. TLR10 has been poorly studied in chronic inflammatory disorders, and its clinical relevance in rheumatoid arthritis (RA) is as yet unknown. We aimed at identifying TLR10 variants within all coding regions of the gene in patients with RA as well as studying their functional and clinical significance.

**Methods:**

TLR10 gene variants were studied by performing sequencing of 66 patients with RA and 30 control subjects. A selected variant, I473T, was then analyzed in 1654 patients and 1702 healthy control subjects. The capacity of this TLR10 variant to modify the transcriptional activity of nuclear factor kappa-light-chain-enhancer of activated B cells (NFkB) was determined by using a luciferase reporter assay and analyzing the expression of NFkB target genes by quantitative polymerase chain reaction. Differences between groups were analyzed by using the Mann-Whitney *U* test and the unpaired two-tailed Student’s *t* test.

**Results:**

We detected ten missense variants in the TLR10 gene and focused on the I473T substitution based on allele frequencies and the predicted functional impact. I473T variant is not associated with susceptibility to RA, but it significantly correlates with erosive disease in patients seropositive for antibodies to citrullinated protein antigens (*p* = 0.017 in the total cohort and *p* = 0.0049 in female patients) and with a lower response to infliximab treatment as measured by the change in Disease Activity Score in 28 joints (*p* = 0.012) and by the European League Against Rheumatism criteria (*p* = 0.049). Functional studies showed that TLR10 reduced activation of the NFkB inflammatory pathway in hematopoietic cells, whereas the I473T variant lacked this inhibitory capacity. Consistently, after exposure to infliximab, cells expressing the I437T variant showed higher NFkB activity than cells carrying wild-type TLR10.

**Conclusions:**

A TLR10 allelic variant, I473T, has impaired NFkB inhibitory activity and is highly associated with disease severity and low response to infliximab in patients with RA.

## Background

Rheumatoid arthritis (RA) is a systemic autoimmune disease characterized mainly by chronic inflammation of the synovial lining and Th1 activation, which lead to progressive joint destruction. It has been suggested that viruses and bacteria may contribute to initiation or exacerbation of RA by binding to Toll-like receptors (TLRs) [1, 2].

TLRs belong to a family of transmembrane proteins that constitute one of the primary defense mechanisms in infectious and some noninfectious diseases in mammals [3]. Inappropriate activation of TLR-mediated pathways has been implicated in the loss of self-tolerance, leading to autoimmunity and chronic inflammation [4, 5]. TLRs are type I transmembrane glycoproteins with extracellular leucine-rich repeats (LRRs) and an intracellular Toll/interleukin (IL)-1 receptor (TIR) homology domain [6]. Signaling by TLRs involves interaction with TIR domain-containing adaptors, including MyD88, TIR-domain-containing adapter-inducing interferon-β (TRIF), TRIF-related adapter molecule (TRAM), TIR-related adapter protein (TIRAP), and sterile alpha and TIR motif-containing protein 1 (SARM1) [7]. These interactions promote nuclear factor kappa-light-chain-enhancer of activated B cells (NFkB) activation, among other functions. TLR-dependent activation of NFkB drives the production of proinflammatory chemokines, cytokines, and cell adhesion molecules [7, 8]. There is increasing evidence that NFkB is a major, if not the main, transcription factor controlling inflammation [9].

TLR10 remains the only orphan member among the human TLRs because its ligands remain unknown and there are discordant data about its function [10, 11]. The expression of TLR10 has been reported mainly in B cells, dendritic cells, eosinophils, neutrophils, and nonimmune cells such as trophoblasts [11–14]. In mammals, TLR10, TLR1, and TLR6 share a common locus on chromosome 4p14 and are structurally similar to one another [11]. Despite interacting with MyD88 [11], TLR10 differs from other TLRs by the lack of a classic downstream signaling pathway [15]. Phylogeny supports the idea that TLR10 arose before the gene duplication that generated TLR1 and TLR6 [16, 17]. TLR1 and TLR6 are able to form a protein complex with TLR2 and TLR10 [11, 14], although the individual contribution of each protein to the function of the complex is largely unknown.

Genetic variants in TLR family members have been associated mainly with disease susceptibility in patients with RA with variable levels of significance and even discordant results [18–21]. In a previous study, researchers investigated the association between TLR10 variants and RA susceptibility, but no statistical significance was found [20]. Although single-nucleotide variants of the *TLR10* gene have been associated with other autoimmune [22] and tumor [23] diseases, the functional activity of this protein and the clinical significance of its gene variants are still controversial [11, 24].

In this article, we report that TLR10 is able to inhibit NFkB signaling in hematopoietic cells, which may limit the activation of this transcription factor that is involved in many chronic inflammatory disorders, including RA. We analyzed the association of a missense variant of TLR10, I473T, with RA and show that this amino acid substitution in an LRR domain gives rise to a protein lacking the NFkB inhibition activity that is associated with more severe disease and lower response to infliximab.

## Methods

### Samples

In this work, we included two cohorts of patients with RA, a first cohort of 453 unselected patients followed at Hospital Universitario Marques de Valdecilla (Santander, Spain) and Hospital Universitario La Paz (Madrid, Spain), and a second one of 1201 patients recruited by the Immune-Mediated Inflammatory Disease Consortium (Spain) [25]. Clinical information, including demographic data, disease characteristics, and treatments, are summarized in Table 1. All patients were diagnosed according to the American College of Rheumatology classification criteria [26]. As a control population, 1702 healthy individuals from the same genetic background were also genotyped [27]. All control individuals had been screened for the presence of an autoimmune disease or a family history of autoimmune disorders, and excluded in case of a positive result.

**Table 1:** Main features of two cohorts of patients with rheumatoid arthritis

	Cohort I (*n* = 453)	Cohort II (*n* = 1201)
Females, %	73.1	76.8
Mean age, years	65.6 ± 14.5^a^	46.5 ± 14.5^a^
Duration of follow-up, months	123.8 ± 91.5^a^	171.6 ± 123.6^a^
Extraarticular manifestations, %	22.2	–
Presence of joint damage, %	–	100
RF-positive, %	64.8	74.6
ACPA-positive, %	62.2	74.1
Number of previous DMARDs	2.2 ± 1.5^a^	1.7 ± 1.5^a^
Number of previous biologic therapies	1.8 ± 1.2^a^	0.6 ± 0.9^a^

*Abbreviations: RF* Rheumatoid factor, *DMARDs* Disease-modifying antirheumatic drugs, *ACPA* Antibodies to citrullinated protein antigens

^a^Mean ± SD

### Cell lines

K562 and U937 cells were maintained in RPMI 1640 medium (Life Technologies, Paisley, UK) supplemented with 10 % FBS (Lonza, Verviers, Belgium). The medium was replaced every 2–3 days.

### Sequencing analysis

By next-generation sequencing (NGS), the coding exons and flanking regions of the *TLR10* gene were sequenced in 66 selected patients with severe RA, rheumatoid factor and/or antibodies to citrullinated protein antigens (ACPA) positivity, erosive disease, and resistance to at least one disease-modifying antirheumatic drug, as well as in 30 healthy control subjects. DNA libraries were processed for hybrid enrichment using a custom Nimblegen SeqCap EZ design (Roche Sequencing, Basel, Switzerland) containing the coding sequences of *TLR10*. Then, double-barcoded libraries were sequenced by using a MiSeq NGS platform (Illumina, San Diego, CA, USA). Sequencing reads were aligned against the human reference genome (hg19) using BWA [28] with the default parameters. Several tools (SAM, GATK, Picard) for manipulating alignments, including sorting, merging, and indexing the BAM files, were used. Single-nucleotide variant and indel calling was performed using GATK Unified Genotyper [29]. Variants were annotated using snpEff, and association studies were performed using Plink software [30].

For Sanger sequencing, DNA was extracted from whole blood by using the QIAamp DNA blood kit (QIAGEN, Hilden, Germany) and amplified with primers for human TLR10: 5′-CATGGCCAGAAACTGTGGTC-3′ and 5′-ACCATCCAACCATCATGACC-3′. Sequence analysis of amplified fragments was carried by using a genetic analyzer (Applied Biosystems, Foster City, CA, USA).

The possible potential impact of an amino acid change on the encoded protein functions was assessed by using two different genomic programs: Sorting Intolerant from Tolerant (SIFT), which uses evolutionary information from homologous proteins [31], and SNPs3D, based on both the structure and a sequence-based method that employs a multiple sequence alignment to build a sequence profile [32].

### Single-nucleotide polymorphism analysis

TLR10 variant I473T (rs11466657) was analyzed in an independent cohort of 1201 patients with RA and 1493 healthy control subjects using the TaqMan® genotyping platform (assay identifier C_25643390_30; Applied Biosystems). All polymerase chain reactions (PCRs) and endpoint fluorescence readings were performed using an ABI PRISM 7900 HT sequence detection system (Applied Biosystems). The genotyping error was estimated by genotyping 20 % of samples in duplicate (error <1 %) [33].

### Transfections and gene reporter assays

TLR10 complementary DNA (cDNA) was cloned into a pCMV6 vector (OriGene, Rockville, MD, USA). The I473T variant was generated by site-directed mutagenesis using the QuikChange mutagenesis kit (Agilent Technologies, Santa Clara, CA, USA) with the following primers: 5′-GGCCTTACGAGAACTAAATACTGCATTTAATTTTCTAACTGATC-3′ and 5′-GATCAGTTAGAAAATTAAATGCAGTATTTAGTTCTCGTAAGGCC-3′. The modified insert was sequenced to verify the mutation. K562 and U937 cells were cotransfected with 2 μg of wild-type or mutant TLR10 constructs, 1 μg of reporter plasmid pBVIx-Luc, containing six tandem repeats of the NFkB recognition sites within the promoter region linked to the luciferase gene [34] and 0.2 μg of respiratory syncytial virus-β-galactosidase plasmid (pRSV-β-gal) by using Lipofectamine 2000 reagent (Sigma-Aldrich, St. Louis, MO, USA). After 24 h of transfection, cell viability was greater than 80 %. Then, K562 and U937 cells were incubated with 10 ng/ml and 20 ng/ml tumor necrosis factor-α (TNFα) in the presence or absence of 200 μg/ml infliximab, and, after 24 h, cell extracts were prepared and analyzed to measure relative luciferase activity by using a dual-light reporter gene assay system (Applied Biosystems). Results were normalized for transfection efficiency with values obtained with pRSV-β-gal.

### Expression analyses of NFkB target genes

To assess the expression of individual genes, cDNA was generated and amplified by using primers for human TNFα (5′-CAATGGCGTGGAGCTGAGAG-3′ and 5′-GGCTGATGGTGTGGGTGAGG-3′), chemokine (C-C motif) ligand 2 (CCL2) (5′-CTCGCTCAGCCAGATGCAAT-3′ and 5′-GTCTTCGGAGTTTGGGTTTGC-3′), TNF-related apoptosis-inducing ligand (TRAIL) (5′-GAGCTGAAGCAGATGCAGGAC-3′ and 5′-TGACGGAGTTGCCACTTGACT-3′), and β-actin (5′-GCTGCCTCAACACCTCAAC-3′ and 5′-GATGGAGTTGAAGGTAGTTTCGTG-3′). Quantitative real-time PCR was performed using the ABI PRISM 7000 Sequence Detection System (Applied Biosystems). The ratio of the abundance of differentiation markers to that of β-actin transcripts was calculated as 2*n*, where *n* is the threshold cycle value of β-actin minus the threshold cycle value of the corresponding messenger RNA (mRNA) and normalized by the value of the sample with the lowest expression level of these genes. The specificity of the desired PCR products was determined by performing melting curve analysis.

### Statistical analysis

All statistical analyses were performed using the SPSS 20 software program (IBM, Armonk, NY, USA) and the R statistical software package (version 3.2.0). Differences in categorical variables between groups of patients were compared using Fisher’s exact test. Statistical significance between groups in in vitro analyses was determined by using an unpaired, two-tailed Student’s *t* test or the Mann-Whitney *U* test. The significance level was set at *p* < 0.05. Genetic association analyses were performed using linear and logistic regression models. The significance of the β regression coefficient was determined using the Wald test. As described previously, covariates in these genetic analyses included the years of disease evolution and the basal Disease Activity Score in 28 joints (DAS28) for the association of rs11466657 genotype with the level of joint damage [33] and the change in DAS28 at 12 weeks [35], respectively.

## Results

### I473T variant is associated with disease severity and treatment response in patients with RA

**Fig. 1:**
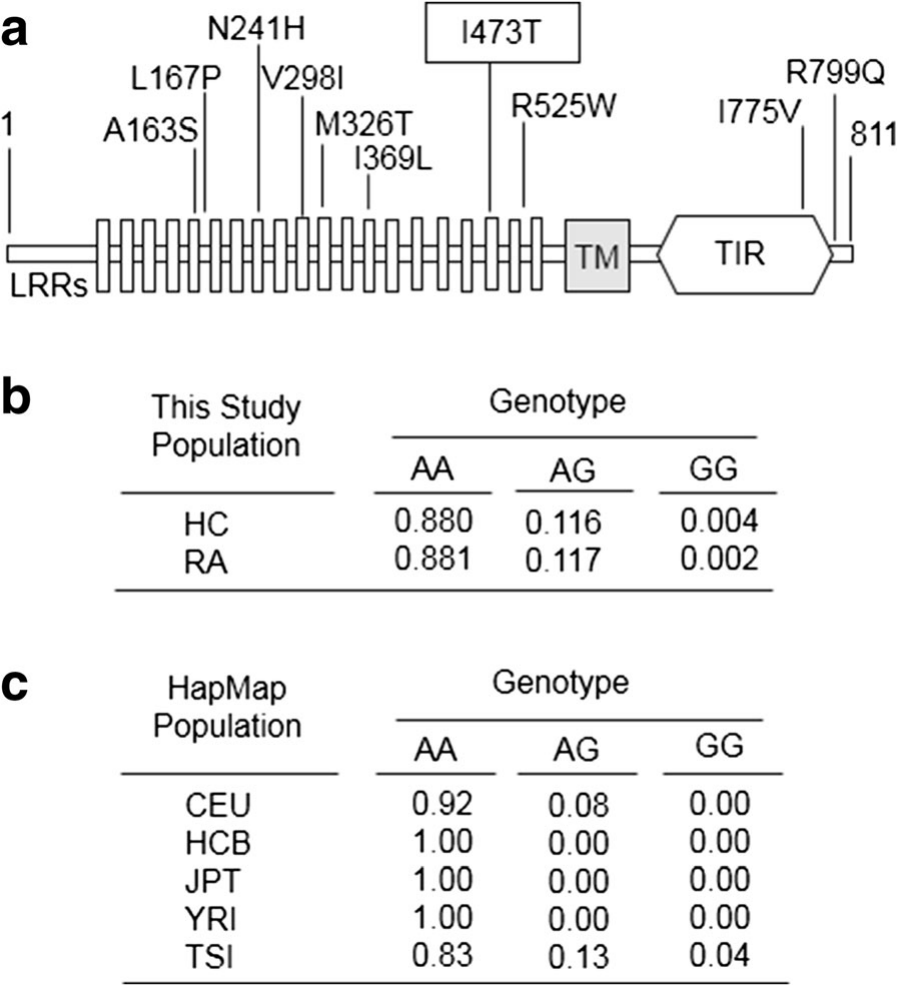
*TLR10* gene variants. **a** Schematic representation of TLR10 protein that illustrates the position of the missense variants identified by next-generation sequencing. **b** Genotype distribution of the I473T variant in the independent cohort of 1493 healthy control subjects (HC) and 1201 patients with rheumatoid arthritis (RA). **c** TLR10 genotype frequencies in HapMap populations. The following population samples were included: CEU (Utah residents with Northern and Western European ancestry from the Centre d’etude du Polymorphisme Humain [CEPH] collection), HCB (Han Chinese in Beijing), JPT (Japanese in Tokyo, Japan), YRI (Yoruba in Ibadan, Nigeria), TSI (Toscani in Italy). *LRR* Leucine-rich repeats, *TLR* Toll-like receptor, *TM* Transmembrane domain, *TIR* Toll/interleukin-1 receptor domain

The function of TLR10 is controversial, and its association with RA has been poorly studied. In order to assess whether TLR10 variants contribute to modifying the course of the disease in patients with RA, we sequenced the coding exons of the *TLR10* gene in 66 selected patients with RA and 30 healthy control subjects. After filtering bases having at least 30× sequence coverage, 16 variants were identified (Table 2). The power to detect genetic effects depends to a great extent on the minor allele frequency (MAF) of the risk allele tested. Ten of the sixteen variants filtered corresponded to missense changes, which are located mainly in LRR domains (Fig. 1a); of these, only two, I473T and L167P, were predicted by using the SIFT and SNPs3D programs to have a functional impact on the protein. The MAF of the L167P variant was less than 0.1 %, whereas the MAF of I473T was about 9 %. Thus, we decided to focus on the I473T variant for clinical association studies.

**Table 2:** Sixteen allele variants of the *TLR10* gene found by next-generation sequencing in control and rheumatoid arthritis populations

Reference SNP ID	Reference allele	Alternative allele	MAF	*p* Value	AA change	Functional class	SIFT prediction	SNPs3D prediction
rs10776482	A	G	0.29	0.2306	D809	Silent	nr	nr
rs4129008	C	T	0.01	0.5652	R799Q	Missense	−	−
rs4129009	T	C	0.271	0.1889	I775V	Missense	−	−
rs10776483	A	G	0.302	0.3296	H724	Silent	nr	nr
rs11466658	G	A	0.026	0.1599	R525W	Missense	−	−
rs11466657	A	G	0.089	0.04327	I473T	Missense	+	+
rs11096955	T	G	0.422	0.07296	I369L	Missense	−	−
rs11096956	C	A	0.292	0.3918	P344	Silent	nr	nr
rs11466653	A	G	0.026	0.5824	M326T	Missense	−	nr
rs11466652	T	C	0.13	0.1403	K303	Silent	nr	nr
rs11466651	C	T	0.026	0.5824	V298I	Missense	−	−
rs11096957	T	G	0.438	0.015	N241H	Missense	−	−
rs11466650	A	G	0.0005	0.137	L167P	Missense	+	+
rs11466649	C	A	0.026	0.5824	A163S	Missense	−	−
rs10856837	C	T	0.026	0.5824	T37	Silent	nr	nr
rs10856838	A	T	0.146	0.5813	I13	Silent	nr	nr

*Abbreviations: MAF* Minor allele frequency, *+* Damaging change, *−* No damaging change, *nr* No records, *SNP* Single-nucleotide polymorphism

**Fig. 2:**
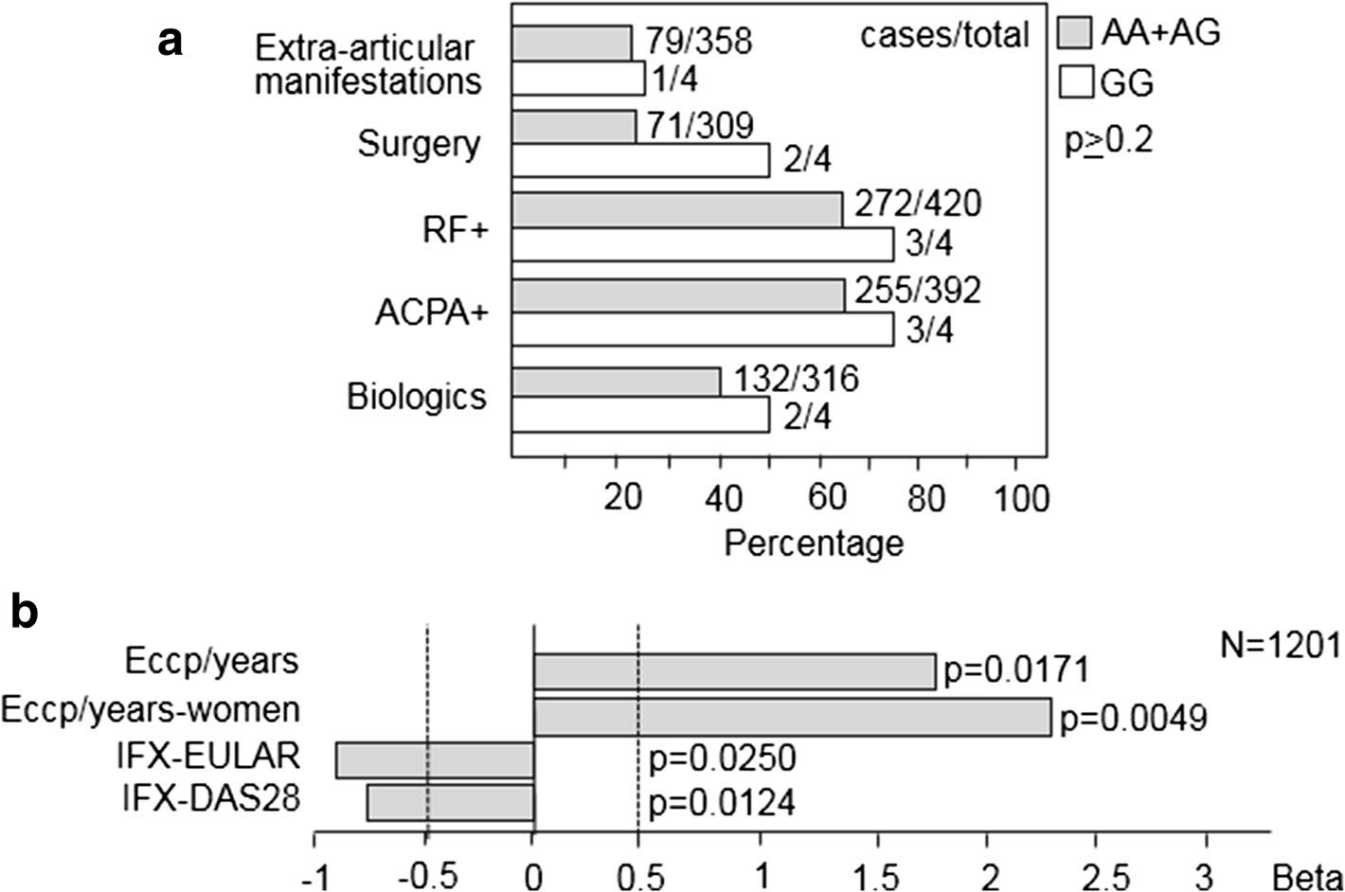
Association of the I473T variant with disease severity in patients with RA. **a** Frequency of clinical parameters associated with severity in AA + AG and GG genotypes in a cohort of 453 patients. Differences were analyzed with the Fisher’s exact test. *RF* ^*+*^ Positive for (high levels of) rheumatoid factor, *ACPA* ^*+*^ Seropositive for antibodies to citrullinated protein antigens. **b** Disease severity associated to the G allele in 1201 patients with RA. The β value (regression coefficient), used to evaluate the effect of I473T variant (genetic risk), was estimated for a number of clinical findings. A positive regression coefficient means that the variant increases risk. *Dotted lines* indicate cutoff values. Clinical parameters were categorized as binary variables. Significance of the β value was determined by using the Wald test. *Eccp/years* Association with erosions in ACPA^+^ patients including the years of disease evolution; *Eccp/years-women* Association with erosions in ACPA^+^ female patients including the years of disease evolution, *IFX-EULAR* Association with response to infliximab (European League Against Rheumatism response criteria: moderate/good vs none), *IFX-DAS28* Association with change in Disease Activity Score in 28 joints in patients treated with infliximab

First, we studied a population of 453 patients and found that the genotype frequencies (86.6 % AA, 11.9 % AG, 1.5 % GG) were not significantly different from those in a population of 209 control subjects (84.5 % AA, 14.3 % AG, 1.2 % GG). This result was confirmed by analyzing the largest genome-wide association study (GWAS) meta-analysis performed to date, which included 29,880 RA cases and 73,758 control subjects [36]. Using the available summaries for the GWAS data in the Caucasian European population, we found no association between the I473T variant and RA susceptibility (*p* = 0.26). We then analyzed a number of clinical findings in the patient population associated with disease severity, including the presence of typical RA autoantibodies, extraarticular manifestations, and the need for specific biologic treatments and surgery. Although differences between AA + AG and GG genotypes did not reach statistical significance (*p* ≥ 0.2), all five clinical criteria showed the same tendency associated with more severe disease only in GG genotype carriers (Fig. 2a). To strengthen this observation, we studied a larger cohort of 1201 patients with RA and 1493 healthy control subjects. As previously observed, the genotype distribution was found to be similar in both patient and control populations (Fig. 1b), which is consistent with the genotypes described for the European population in the HapMap database (Fig. 1c). Interestingly, as shown in Fig. 2b, the I473T variant is highly associated with erosive disease in ACPA-positive patients with RA (*p* = 0.017 in the total cohort and *p* = 0.0049 in female patients) and with a lower response to infliximab treatment as measured by the change in DAS28 score (*p* = 0.012 in the total cohort) as well as by the European League Against Rheumatism response criteria (*p* = 0.025 in the total cohort), which indicate that this variant is represented in a group of patients with more severe disease.

### Variant I437T modifies the NFkB regulatory capacity of TLR10

**Fig. 3:**
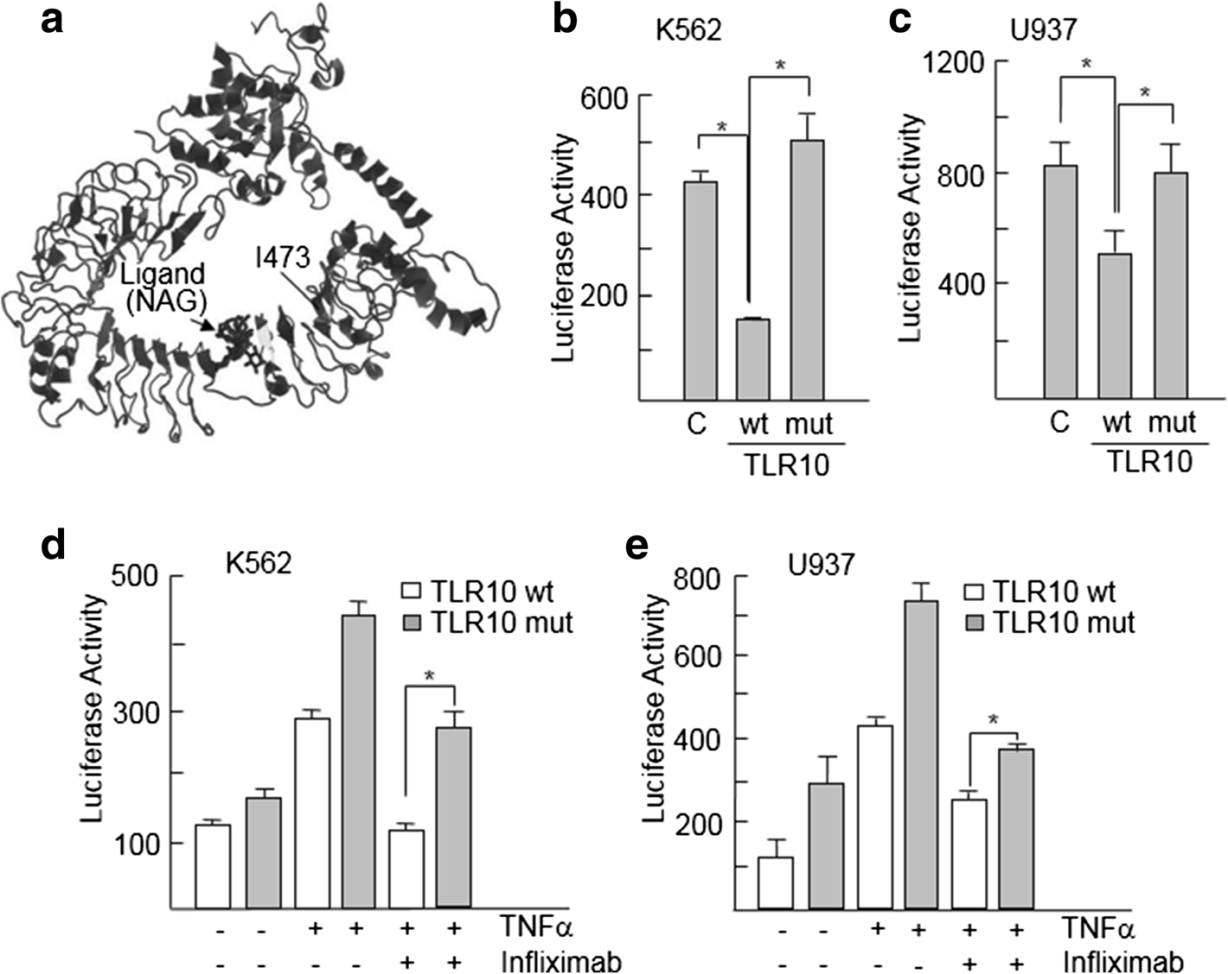
Regulation of nuclear factor kappa-light-chain-enhancer of activated B cells (NFkB) transcriptional activity by the I473T variant. **a** Three-dimensional structure of Toll-like receptor 10 (TLR10) visualized by using the Jmol viewer showing the location of residue I473. *NAG* 2-(acetylamino)-2-deoxy-β-d-glucopyranose. **b** and **c** K562 and U937 cells were cotransfected with wild-type (wt) TLR10 or its mutated variant (mut) and a reporter vector containing NFkB-responsive sequences. Cells were stimulated with 10 ng/ml tumor necrosis factor-α (TNFα) for 24 h, and then cell extracts were prepared and analyzed for luciferase activity. **d** and **e** K562 and U937 cells transfected with the indicated TLR10 variants were treated with TNFα in the presence or absence of infliximab, and luciferase activity was analyzed. Histograms show the mean + SD of three independent experiments. *p < 0.05

**Fig. 4:**
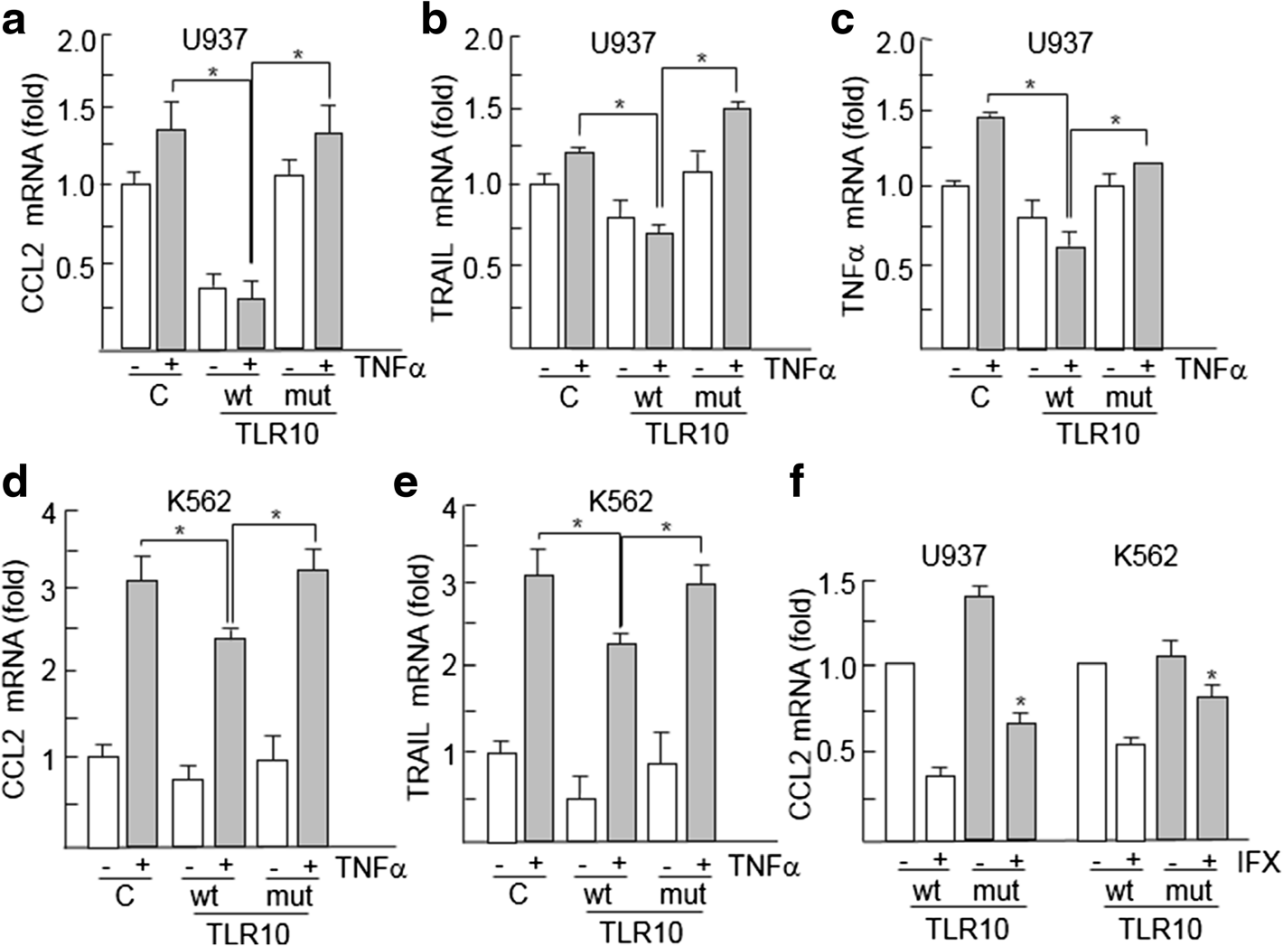
Regulation of the levels of nuclear factor kappa-light-chain-enhancer of activated B cells (NFkB) target genes by the I473T variant. U937 and K562 cells were transfected with Toll-like receptor 10 (TLR10) (wild type [wt] or mutated variant [mut]) and then treated or not with 20 ng/ml tumor necrosis factor-α (TNFα) for 24 h. The expression of NFkB target genes chemokine (C-C motif) ligand 2 (*CCL2*) (**a**, **d**), TNF-related apoptosis-inducing ligand (*TRAIL*) (**b**, **e**), and *TNFα* (**c**) were determined by performing quantitative real-time polymerase chain reactions. **f** Cells transfected with the indicated variants were treated with TNFα in the presence or absence of infliximab, and, 24 h later, the messenger RNA (mRNA) levels of CCL2 were determined. β-actin was used for normalization. *C* Cells transfected with empty vector as an additional control. Histograms show the mean ± SD of three independent experiments. *p < 0.05

On the basis of a three-dimensional structure model visualized by using the Jmol viewer (Fig. 3a), we found that position 437 is within a β-strand in the LRR18 domain and is occupied by an isoleucine, a highly hydrophobic amino acid, buried inside the protein core. This position is occupied by hydrophobic amino acids in TLR10 (Ile), TLR2 (Ile), and TLR1 (Val), which belong to the same subfamily of TLRs that are located at the cell membrane and dimerize with each other [37]. In the TLR10 variant, isoleucine is substituted by threonine, a polar amino acid that may participate in hydrogen bonds and is usually located at the protein surface. LRR domains provide an outstanding framework for achieving diverse protein interactions. Thus, this structural change decreases hydrophobic contacts and may alter functionally relevant protein-protein interactions. TLR family members are known to be regulators of the NFkB activity, a key pathway in inflammation. To experimentally prove the functional consequences of the I437T variant on the activation of NFkB, we introduced the codon-altering nucleotide in a construct containing TLR10 cDNA by site-directed mutagenesis and studied the capacity of this variant to modify the NFkB transcriptional activity. As shown in Fig. 3b and c, wild-type TLR10 downregulates the transcriptional activity of NFkB after stimulation with an inflammatory cytokine, TNFα, in K562 and U937 hematopoietic cell lines, which are widely used to study NFkB activation [38]. However, the I437T substitution blocks the inhibition capacity of TLR10. In order to translate the reduced response to infliximab in patients to our in vitro model, we stimulated cells with TNFα in the presence or absence of infliximab. Consistently, cells expressing the I437T variant had a higher level of NFkB activity remaining after treatment with infliximab than cells expressing the wild-type variant (Fig. 3d, e). Next, we confirmed this result by analyzing the expression of NFkB target genes. The expression levels of CCL2, TRAIL, and TNFα were downregulated in the presence of wild-type TLR10 following stimulation of the NFkB pathway with TNFα (Fig. 4a–e). In line with our previous results, downregulation of NFkB target genes was abrogated when U937 and K562 cells where transfected with the variant-containing construct and cultured with TNFα (Fig. 4a–e). Moreover, gene expression also confirmed that the TLR10 variant slightly reduced the response of TNFα-activated cells to infliximab (Fig. 4f). Overall, the in vitro data show that the I437T variant abrogates the NFkB inhibition activity of TLR10 and displays a weak but consistent resistance to the anti-inflammatory effects of infliximab.

## Discussion

Deciphering the mechanisms that regulate the activity of NFkB is of major importance for understanding the response to inflammatory stimuli. TLRs have been suggested to be implicated in responses to pathogens in RA [39]. Nowadays, TLR10 is the only member of the TLR family without a well-defined biological function. In this study, we show that TLR10 displays NFkB inhibitory activity in hematopoietic cells following treatment with inflammatory stimuli. Researchers in several studies have described how TLR10 acts as a proinflammatory receptor activating NFkB signaling [11, 40]. Another study showed that TLR10 fails to activate typical TLR-induced signaling, including transcriptional activation mediated by NFkB or interferon-β [15]. Recently, it has been shown that TLR10 is a modulator with mainly inhibitory effects [24]. In line with this, blocking TLR10 by antagonistic antibodies enhances proinflammatory cytokine production [10]. Also, evidence has been provided indicating that TLR10 induces apoptosis through activation of caspase-3 [18], which is consistent with the NFkB inhibitory activity of TLR10 because NFkB is an antiapoptotic transcription factor. These contradictory results may be due to the complexity of the modulatory effects of TLR10 that involve several mechanisms, including competition for ligands, heterodimerization with other TLRs, and activation of different signaling pathways.

On the basis of this information, the cell model used to study TLR10 may be relevant to evaluation of the functional activities of this receptor and should be taken into consideration when comparing different studies regarding the activities of TLR10. Researchers in several studies have investigated the role of TLR polymorphisms in RA, although the results obtained are controversial [19–21, 41]. However, TLR10 genetic variants have not yet been associated with RA, but they have been associated with susceptibility to infectious and inflammatory diseases such as extrapulmonary tuberculosis and asthma [42, 43]. We found that the I473T variant significantly associates with the presence of ACPA and erosive disease, both in the total RA cohort and in female patients. It is well established that TNFα inhibitors help control disease activity. However, not all patients respond adequately to initial anti-TNFα treatment [44]. In line with this, we have demonstrated that patients with the I437T variant showed a significant lower response to infliximab, and this effect was also demonstrated in vitro. Thus, this TLR10 variant may be a good candidate marker of response to infliximab or other anti-TNFα treatment in patients with RA, which should be validated by replicating this study in other populations with different genetic backgrounds. Although there were no differences in genotype distribution between patients with RA and healthy control subjects, our data show that a TLR10 genetic variant selects a group of patients with a more severe and refractory disease. This variant has been described only in previous work associated with a decreased risk of meningioma [45]. The I473T variant leads to a reduced capacity of TLR10 to inhibit activation of NFkB in response to inflammatory stimuli. This effect may be explained by the amino acid change decreasing the hydrophobicity of an LRR domain, which may alter the interaction with TLR proteins needed for TLR10 signaling [37].

## Conclusions

Overall, our observations identify an allelic variant, I473T, in TLR10 that is significantly associated with more severe disease. Our data also indicate that the TLR10 variant lacks the capacity to inhibit NFkB transcriptional activity in hematopoietic cells and reduces the clinical and biological response to infliximab, which support the association between the I473T variant and disease outcome in patients with RA.

## Abbreviations

ACPA: Antibodies to citrullinated protein antigens
CCL2: Chemokine (C-C motif) ligand 2
cDNA: Complementary DNA
CEU: Utah residents with Northern and Western European ancestry from the Centre d’etude du Polymorphisme Humain (CEPH) collection
DAS28: Disease Activity Score in 28 joints
DMARD: Disease-modifying antirheumatic drug
GWAS: Genome-wide association study
HC: Healthy control subjects
HCB: Han Chinese in Beijing
IFX-EULAR: Association with response to infliximab based on European League Against Rheumatism response criteria
IFX-DAS28: Association with change in Disease Activity Score in 28 joints in patients treated with infliximab
JPT: Japanese in Tokyo, Japan
LRR: Leucine-rich repeat
MAF: Minor allele frequency
mRNA: Messenger RNA
NAG: 2-(Acetylamino)-2-deoxy-β-d-glucopyranose
NFkB: Nuclear factor kappa-light-chain-enhancer of activated B cells
NGS: Next-generation sequencing
nr: No records
PCR: Polymerase chain reaction
pRSV-β-gal: Respiratory syncytial virus-β-galactosidase plasmid
RA: Rheumatoid arthritis
RF: Rheumatoid factor
SARM1: Sterile alpha and TIR motif-containing protein 1
SIFT: Sorting Intolerant from Tolerant
SNP: Single-nucleotide polymorphism
TIR: Toll/interleukin-1 receptor
TIRAP: TIR-related adapter protein
TLR: Toll-like receptor
TM: Transmembrane domain
TNF: Tumor necrosis factor
TRAIL: TNF-related apoptosis-inducing ligand
TRAM: TRIF-related adapter molecule
TRIF: Toll/interleukin-1 receptor domain-containing adapter-inducing interferon-β
TSI: Toscani in Italy
wt: Wild type
YRI: Yoruba in Ibadan, Nigeria
